# Guillain-Barré syndrome following intracranial hemorrhage: a systematic review of case reports

**DOI:** 10.3389/fneur.2026.1789597

**Published:** 2026-06-17

**Authors:** Sai Krishna Vallamchetla, Sai Kumar Reddy Pasya, Gary Gronseth, Long Davalos

**Affiliations:** 1 Department of Neurology, Mayo Clinic, Jacksonville, FL, United States; 2 Department of Neurology, University of Kansas Medical Center, Kansas City, KS, United States

**Keywords:** acute inflammatory demyelinating polyneuropathy, Guillain-Barré syndrome, intracerebral hemorrhage, intracranial hemorrhage, subarachnoid hemorrhage, axonal neuropathy, polyneuropathy

## Abstract

**Introduction:**

Guillain-Barré syndrome (GBS) is an autoimmune polyradiculopathy linked to various triggers. Recently, several case reports described GBS development following intracranial hemorrhage (ICH), posing diagnostic and therapeutic challenges.

**Methods:**

PubMed and EMBASE were searched from inception till February 2023 for case reports. Two reviewers independently screened the articles and extracted data using a standardized form. The extracted data were then analyzed narratively. Quality was assessed with the JBI Checklist for Case Reports.

**Results:**

Twenty-three cases from 11 countries were identified (14 males, mean age of 60.1 years). Onset of GBS occurred after a median of 9 days (range 2–24) post-ICH, with intracerebral hemorrhage being the most common antecedent (39.1%), followed by subarachnoid (26.1%) and subdural hemorrhage (21.7%). Electrophysiology showed predominantly axonal variants, while CSF consistently demonstrated albuminocytologic dissociation with positive antiganglioside antibodies in five cases. The clinical course was severe, marked by rapid progression to nadir (median 2.5 days), profound functional impairment (average Hughes Functional Grading Scale: 4.57 ± 0.66), and a high rate of respiratory failure requiring mechanical ventilation (78.3%). Axonal variants were associated with higher rates of mechanical ventilation and poorer outcomes than demyelinating forms. Among the 21 patients who received immunomodulatory treatment, 3 (14.3%) showed complete recovery, 13 (61.9%) had partial recovery, 3 (14.3%) had a poor recovery, and 2(9.5%) died at last follow up.

**Conclusion:**

GBS may complicate recovery after ICH and appears to present with an aggressive course, particularly in axonal variants. Clinicians should consider GBS when patients with ICH develop progressive, symmetrical weakness unexplained by the initial brain injury, as prompt initiation of immunotherapy may improve outcomes.

## Introduction

Guillain-Barré syndrome (GBS) is an acute autoimmune disorder of the peripheral nervous system characterized by progressive, bilateral, and symmetrical limb weakness, with generalized hyporeflexia or areflexia, which maybe further complicated by respiratory failure and autonomic dysfunction (1). Although the etiology of GBS is not fully understood, it is widely acknowledged that various infectious agents, such as *Campylobacter jejuni*, *Mycoplasma pneumoniae*, Cytomegalovirus, Epstein–Barr virus and Zika Virus, can elicit an immune response that ultimately leads to the development of GBS (2, 3). Other less common, non-infectious antecedent events, such as surgery, pregnancy, immune checkpoint inhibitor therapy, trauma, bone marrow transplantation, and systemic lupus erythematosus, have also been reported (2, 4–8).

Several recent case reports have described the development of GBS following intracranial hemorrhage (ICH), though the relationship between these conditions remains poorly understood (9). Despite their distinct pathophysiological mechanisms, both conditions are neurological emergencies, and their coexistence poses a unique diagnostic and therapeutic challenge to healthcare providers. To date, the literature has been limited to individual case reports, resulting in a lack of comprehensive data on the clinical presentation, diagnosis, and management of this rare association. To address this gap, we conducted a systematic review of the literature to analyze and synthesize the existing data on GBS occurring after ICH, with the goal of providing a clear understanding of its clinical features, diagnosis, and management.

## Methods

### Study design

This systematic review was conducted following the Preferred Reporting Items for Systematic Reviews and Meta-Analyses (PRISMA) guidelines (10).

### Search strategy

Two electronic databases (EMBASE and PubMed) were searched from inception until February 2023. The search strategy included a combination of MeSH terms and keywords related to GBS and Intracranial hemorrhage.

### Study selection

Two independent reviewers screened the abstracts of all articles identified by the search strategy (11). Full-text articles were retrieved for all abstracts that met the inclusion criteria or where there was any uncertainty. Two independent reviewers then assessed the full-text articles for inclusion in the systematic review. Disagreements were resolved by consensus or by a third reviewer. We also manually searched the reference lists of included studies and relevant review articles to identify additional studies. The Joanna Briggs Institute (JBI) Critical Appraisal Checklist for Case Reports was used to assess the quality of the included studies (12).

This systematic review included case reports of patients with a diagnosis of GBS following intracranial hemorrhage, published in peer-reviewed journals were included in the review. To be eligible, cases had to meet the National Institute of Neurological Disorders and Stroke (NINDS) diagnostic criteria for Guillain–Barré syndrome. For each eligible case report, two authors independently reviewed the published clinical, cerebrospinal fluid, and electrophysiological data to verify that the NINDS criteria were fulfilled. Excluded from the consideration were cases of GBS not related to intracranial hemorrhage, review articles, meeting abstracts as well as studies that are not published in peer-reviewed journals. Studies that are not in English are translated.

### Data extraction

Two independent reviewers extracted data using a standardized form, including study and patient characteristics, clinical characteristics, treatment, and outcomes.

### Data synthesis

Because the available evidence consisted solely of heterogeneous case reports, we did not perform a formal quantitative meta-analysis. Instead, we used a narrative synthesis approach. Data from each case were entered into a standardized extraction sheet and collated into summary tables. We grouped variables *a priori* into the following domains: (1) study and patient characteristics, (2) type and severity of hemorrhage, (3) clinical features of GBS, (4) investigations, and (5) treatment and outcomes. Within each domain, we summarized data descriptively (frequencies, proportions, means or medians with ranges, as appropriate) and described patterns and differences between subgroups (e.g., traumatic vs. spontaneous hemorrhage, axonal vs. demyelinating variants) in the text. Quality assessment using the JBI Critical Appraisal Checklist for Case Reports was summarized narratively to contextualize the certainty of the evidence but was not used as an exclusion criterion. The JBI checklist comprises eight items that assess the completeness and clarity of reporting of patient demographics, clinical history and presentation, diagnostic work-up, therapeutic interventions, post-intervention course, adverse events, and key take-home messages. Each item is rated as yes, no, unclear, or not applicable; for each case report we counted the number of items rated ‘yes’ (range 0–8) to provide an overall indicator of reporting quality.

## Results

**Figure 1:**
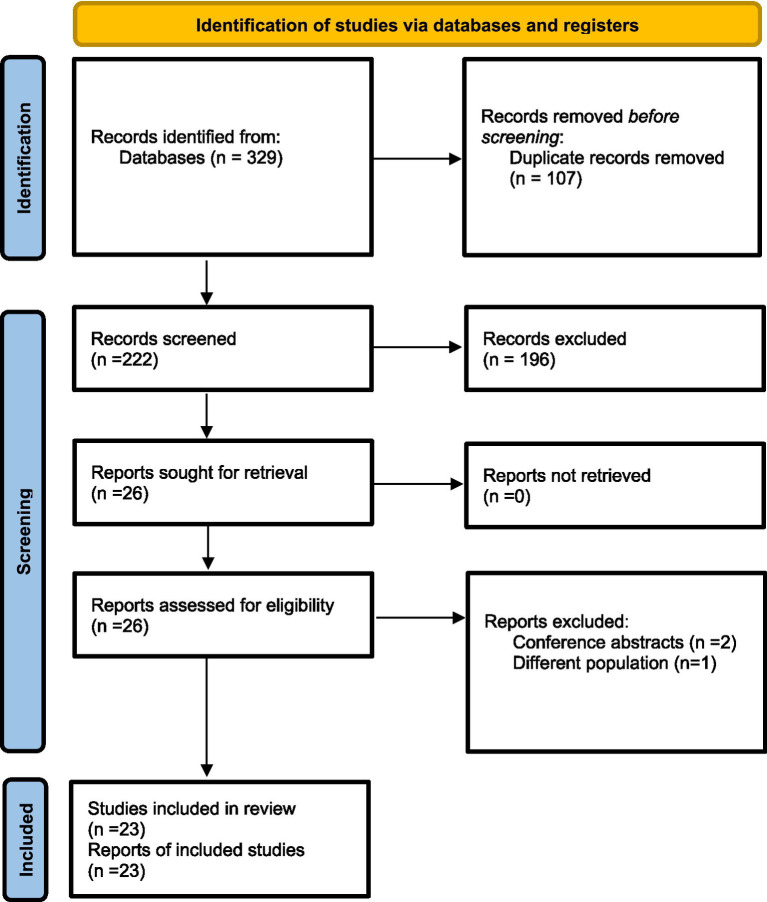
Flowchart of study selection.

This Systematic review includes 23 case reports (Figure 1), and the data extracted from these studies are presented in Tables 1–3.

**Table 1:** Study characteristics.

S. no	First author & year	Country	Age (Yr)	Gender	Type of hemorrhage	Location of hemorrhage	Traumatic/spontaneous
1	Liu et al. (2022) (40)	China	62	M	Intracerebral	Right temporal and insular lobes	Spontaneous
2	Abdulmana et al. (2021) (14)	Saudi Arabia	74	F	Intracerebral	Hemorrhagic transformation in right MCA zone	Spontaneous
3	Abdulmana et al. (2021) (14)	Saudi Arabia	61	M	Intracerebral	Right frontoparietal (midline shift +)	Spontaneous
4	Yonekura et al. (2018) (18)	Japan	74	M	Subdural & Subarachnoid	Bilateral parietooccipital areas	Traumatic
5	Duncan et al. (1987) (41)	UK	61	M	Subdural	Right subdural	Traumatic
6	Yilmaz et al. (2020) (42)	Turkey	41	M	Subdural	Left temporo-occipital	Traumatic
7	Connor et al. (2021) (27)	Australia	64	M	Intracerebral	Right temporo-occipital	Spontaneous
8	Joshi et al. (2019) (43)	Australia	70	F	Intracerebral	Left basal ganglia	Spontaneous
9	Wu et al. (2016) (44)	China	51	F	Subarachnoid	Ambient and posterior interhemispheric cisterns	Spontaneous
10	Wu et al. (2016) (44)	China	68	M	Intracerebral	Hemorrhagic transformation in left MCA zone	Spontaneous
11	Carr et al. (2015) (15)	USA	58	M	Subarachnoid	n/a (mass effect +)	Traumatic
12	Mantero et al. (2013) (16)	Italy	79	F	Intracerebral	Left cerebellum (mass effect +)	Spontaneous
13	Riancho et al. (2013) (45)	Spain	63	F	Subarachnoid	Right frontoparietal region	Spontaneous
14	Battaglia et al. (2013) (46)	France	73	F	Intracerebral	Bilateral temporal and right frontal	Traumatic
15	Yardimci et al. (2009) (47)	Turkey	75	F	Subdural	Bilateral frontal	Traumatic
16	Tan et al. (2010) (48)	Australia	44	M	Subarachnoid	n/a	Spontaneous
17	Rivas et al. (2008) (17)	USA	55	M	Subdural	Left posterior (no mass effect)	Traumatic
18	Freitas et al. (1997) (49)	Brazil	29	M	Subarachnoid	Left cerebral hemisphere	Traumatic
19	Hu et al. (2020) (50)	China	73	F	Intraventricular	Lateral, third, and fourth ventricles	Spontaneous
20	Jia et al. (2017) (52)	China	33	M	Intracerebral	Right basal ganglia	Spontaneous
21	Jia et al. (2017) (52)	China	41	M	Subdural	Left frontal	Traumatic
22	Song et al. (2012) (51)	China	52	F	Pontine	Pontine tegmentum of the brain stem	Spontaneous
23	Hantson et al. (2013) (13)	UK	81	M	Subarachnoid	n/a	Spontaneous

Yr, year; M, male; F, female; UK, United Kingdom; USA, United States of America; MCA, middle cerebral artery; n/a, not available. Hemorrhage location and radiographic features, including mass effect and midline shift, are reported as described in the original case reports when available.

**Table 2:** Clinical characteristics.

S. No	Time interval between hemorrhage and GBS onset (days)	Pre-GBS deficits	Sensory involvement	Areflexia	Autonomic involvement	Respiratory involvement	Hughes Score at onset of disease	Diagnosis of GBS (level of certainty)*
1	8	Left-sided hemiparesis	Yes	Yes	NA	Yes	4	Clinical features+ CSF analysis (Level 1)
2	5	Left hemiparesis and dysarthria	Yes	Yes	Yes	Yes	5	Clinical features+ CSF analysis+ Electrophysiology (Level 1)
3	7	Left-sided weakness and impaired consciousness.	Yes	Yes	NA	Yes	5	Clinical features + CSF analysis + Electrophysiology (Level 1)
4	2	None	Unclear: due to sedation	Yes	NA	Yes	5	Clinical features+ CSF analysis (Level 1)
5	9	None	Yes	Yes	NA	Yes	5	Clinical features+ Electrophysiology (Level 2)
6	14	None	NA	Yes	NA	Yes	5	Clinical features+ CSF analysis+ Electrophysiology (Level 1)
7	11	Diplopia	Yes	NA	NA	Yes	3	Clinical features+ CSF analysis+ Electrophysiology (Level 1)
8	14	Right-sided hemiplegia	Yes	Yes	Yes	Yes	5	Clinical features+ Electrophysiology (Level 2)
9	10	None	NA	Yes	NA	Yes	5	Clinical features+ CSF analysis+ Electrophysiology (Level 1)
10	7	Right-sided hemiplegia	Yes	Yes	NA	Yes	5	Clinical features+ CSF analysis+ Electrophysiology (Level 1)
11	16	None	Yes	Yes	NA	No	5	Clinical features+ CSF analysis+ Electrophysiology (Level 1)
12	11	None	NA	Yes	NA	Yes	4	Clinical features+ CSF analysis+ Electrophysiology (Level 1)
13	3	Proximal tetra paresis, areflexia	NA	Yes	Yes	Yes	4	Clinical features+ CSF analysis+ Electrophysiology (Level 1)
14	7	None	Yes	Yes	NA	No	5	Clinical features+ CSF analysis+ Electrophysiology (Level 1)
15	10	None	NA	Yes	NA	NA	5	Clinical features+ CSF analysis+ Electrophysiology (Level 1)
16	7	None	Yes	Yes	NA	Yes	5	Clinical features+ CSF analysis+ Electrophysiology (Level 1)
17	7	None	NA	Yes	NA	Yes	5	Clinical features+ CSF analysis+ Electrophysiology (Level 1)
18	5	None	NA	Yes	Yes	yes	5	Clinical features+ CSF analysis+ Electrophysiology (Level 1)
19	3	None	NA	Yes	NA	Yes	4	Clinical features+ CSF analysis+ Electrophysiology (Level 1)
20	14	Left hemiparesis	NA	Yes	NA	Yes	4	Clinical features+ CSF analysis+ Electrophysiology (Level 1)
21	14	None	NA	Yes	NA	Yes	5	Clinical features+ CSF analysis+ Electrophysiology (Level 1)
22	21	Quadriparesis, bilateral facial weakness, severe dysarthria	NA	Yes	Yes	NA	4	Clinical features+ CSF analysis+ Electrophysiology (Level 1)
23	21	Comatose at admission	NA	Yes	NA	Yes	3	Clinical features+ CSF analysis+ Electrophysiology (Level 1)

GBS, Guillain-Barré Syndrome; CSF, cerebrospinal fluid; EP, electrophysiology; NA, Not Available or Not Assessed. *Level of diagnostic certainty according to the Brighton criteria.

**Table 3:** Investigations, treatment, and outcomes.

S. no	CSF cells level (<5 cells/ul)	CSF protein level (15–45 mg/dl)	Albumino-cytological dissociation	Tested for *C. jejuni* or other viruses	Antiganglioside antibodies	Neurophysiological studies	Variant	Immunomodulatory treatment	Clinical Outcome
1	25	102	No	No	Yes	NA	AMSAN	IVIG+ methylprednisolone	Poor recovery, LAMA
2	1	370	Yes	No	Yes	NCS: Absent motor and sensory responses from the sural, superficial peroneal, median, and ulnar nerves with stimulation of the four limbs	AMSAN	IVIG	Poor recovery, full ventilator support on tracheostomy for 2 months, Death due to severe dysautonomia
3	10	161.1	No	No	Yes	NCS: demonstrating absent sensory, motor, and F responses from the upper and lower limbs’ nerves	AMSAN	IVIG + PP	Poor recovery in 1 month follow up
4	8	92	No	No	No	NA	NA	IVIG	Partial recovery in 8 weeks follow up
5	NA	NA	NA	No	NA	NCS: Severe generalized predominantly motor demyelinating peripheral neuropathy entirely consistent with the diagnosis of GBS	AIDP	PP	Complete recovery in 5 weeks follow up
6	NA	NA	Yes	Yes	No	NCS: showed prolonged F-wave latencies and evidence of conduction block in both upper and lower extremity motor nerves.	AIDP	IVIG	Complete recovery in 1 month follow up
7	18	148	No	Yes	No	NCS, EMG: no spontaneous activity or activation in response	NA	IVIG	Partial recovery in 3 months follow up
8	NA	NA	NA	No	NA	NCS: Diffuse, predominantly demyelinating polyneuropathy with superimposed proximal axonal degeneration, absent F waves in both upper and lower limbs, and evidence of denervation in the proximal upper limb and chest muscles	AMSAN	IVIG	Partial recovery
9	12	390.2	No	No	Yes	NCS: Decreased occurrence rate of F-waves, spontaneous potential observed in left gastrocnemius, femoral, and anterior tibialis muscle, decreased left tibial motor nerve conduction velocity	AMAN	IVIG	Partial recovery in 1 year follow up
10	8	290.2	No	No	NA	NCS: Motor nerve conduction velocity was decreased, debased amplitude, prolonged latency and decreased motor and sensory nerve conduction velocity.	AIDP	IVIG	Poor recovery in 1 month follow up
11	5	117	Yes	No	Yes	NCS: were significant for prolonged F wave latencies and evidence of conduction block in both upper and lower extremity motor nerves.	AMAN	IVIG	Partial recovery
12	NA	NA	Yes	No	NA	EMG: proximal partial conduction blocks in the lower limbs and significant increase of distal latencies and F wave in the lower limbs.	AIDP	IVIG + PP	Complete recovery in 6 months follow up
13	NA	65	Yes	No	NA	NCS: Demyelinating polyradiculoneuropathy with a frank increase in distal latencies and virtually generalized absence in F waves.	NA	IVIG	Partial recovery
14	NA	NA	Yes	Yes	No	NCS: increase of motor distal latencies and temporal dispersion of motor action potential in four limbs, with F-waves latencies increased in lower limbs, and all sensory responses were abolished.	AMSAN	IVIG	Partial recovery in 3-month follow-up
15	NA	NA	Yes	Yes	No	NCS: Severe generalized, predominantly motor-demyelinating peripheral neuropathy	AIDP	PP	Partial recovery in 3 months follow up
16	NA	182	Yes	Yes	No	NCS: Absent motor and sensory responses, absent blink reflexes, and an absence of spontaneous and voluntary activity on electromyography	Axonal (NOS)	IVIG	Partial recovery in 10 months follow up
17	Xanthochromia+, Normal cell count	NA	Yes	No	No	NCS: Inexcitability of all nerves and fibrillation potentials of all muscles sampled, severe loss of myelinated axons without significant demyelination	NA	PP	Partial recovery in 6 months follow up
18	3	200	Yes	No	NA	EMG: demyelinating pattern	AIDP	Death before treatment was given	No recovery, Death
19	8	107.3	No	No	No	NA	NA	IVIG	No recovery, Death at 1 month follow up
20	4	560	Yes	No	No	NCS: CMAP of the right ulnar nerve significantly decreased; motor nerve conduction velocity (NCV) was close to normal.	AMAN	IVIG	Partial recovery in a 4-month follow-up
21	2	187	Yes	No	NA	NCS: CMAP of the common peroneal nerve on both sides were significantly decreased; F waves were not evoked in either upper or lower limbs; NCV was close to normal.	AMAN	IVIG	Partial recovery in 6 months follow-up
22	Normal cell count	458	Yes	No	No	NCS: markedly reduced compound motor action potential amplitudes of the median (0.1 mV), ulnar (0.2 mV) and peroneal nerves (0.5 mV), whereas distal latencies and motor conduction velocities were normal. The results of sensory conduction studies were completely normal. Fibrillation potentials and positive sharp waves were found in all examined muscles.	AMAN	IVIG	Partial recovery in 1 year follow-up.
23	Normal cell count	122	Yes	No	No	NCS: consistent with a severe axonopathy (absent distal compound muscle action potentials) and mild reduction of conduction velocities.	Axonal form of GBS	No immunomodulatory treatment	No recovery, death at a 7 month follow up due to infectious complications

AIDP, Acute Inflammatory Demyelinating Polyneuropathy; AMAN, Acute Motor Axonal Neuropathy; AMSAN, Acute Motor and Sensory Axonal Neuropathy; *C. jejuni*, *Campylobacter jejuni*; CMAP, Compound Muscle Action Potential; CSF, Cerebrospinal Fluid; EMG, Electromyography; IVIG, Intravenous Immunoglobulin; LAMA, Left Against Medical Advice; NA, Not Available or Not Assessed; NCS, Nerve Conduction Studies; NCV, Nerve Conduction Velocity; PP, Plasmapheresis.

### Demographic characteristics

BS Cases (*n* = 23) were from the China (*n* = 7), Turkey (*n* = 2), USA (*n* = 2), UK (*n* = 2), Australia (*n* = 3), Japan (*n* = 1), France (*n* = 1), Saudi Arabia (*n* = 2), Brazil (*n* = 1), Italy (*n* = 1) and Spain (*n* = 1) (Table 1). Of the 23 patients, 14 were male (60.9%) and 9 were female (39.1%), with a male-to-female ratio of 1.56. The average age was 60.1 ± 14.8 years (median: 62 years; range: 29–81 years). There was a statistically significant difference in age at onset between males and females (mean: 55.1 ± 15.4 vs. 67.8 ± 10.2 years, respectively; *p* = 0.028).

### Type and severity of hemorrhage

Intracerebral hemorrhage was the most common type (39.1%), followed by subarachnoid hemorrhage (26.1%) and subdural hemorrhage (21.7%). Epidural hematoma was not reported, whereas intraventricular hemorrhage and a single case of combined subdural and subarachnoid hemorrhage were relatively rare, each representing 4.3% of cases. There were 10 traumatic and 13 spontaneous hemorrhages. In the traumatic group, subdural hematoma and subarachnoid hemorrhage were most common (4 and 3 cases, respectively), while intracerebral hemorrhage predominated in the spontaneous group (7 cases). Hemorrhage locations were heterogeneous (Table 1), with a clear predominance of supratentorial involvement, including the frontoparietal, temporal, basal ganglia, and middle cerebral artery territories, whereas infratentorial hemorrhages were uncommon, observed in only two cases involving the pontine tegmentum and cerebellar hemisphere.

Details regarding the severity of the initial hemorrhage were heterogeneously reported. Standardized severity grading scales were rarely used, with only one case reporting Fisher Grade IV (13). The initial Glasgow Coma Scale (GCS) was available for 6 of the 23 cases. Among these, 3 patients (50%) presented with a mild brain injury (GCS 13–15), 1 (16.7%) with a moderate injury (GCS 9–12), and 2 (33.3%) with a severe injury (GCS < 9). Radiographic features such as midline shift and mass effect were explicitly documented in only four cases, being present in three (14–16) and absent in one (17), while in several other reports, repeat imaging at the time of GBS symptom onset showed stability or regression of the hemorrhage, indirectly suggesting the absence of progressive mass effect. Surgical intervention for the hemorrhage was required in 6 cases (26.1%). Furthermore, 11 patients (47.8%) had pre-existing neurological deficits from the initial hemorrhage prior to the onset of GBS.

This inconsistency in radiographic reporting limits the ability to systematically assess the contribution of hemorrhage severity and anatomical burden to the neurological outcomes reported in this cohort.

### Clinical features of GBS

Among the 23 patients, the median time to symptom onset following ICH was 9 days (interquartile range, 7) (Table 2). The minimum interval was 2 days (18), and the maximum was 21 days (13, 51), suggesting a considerable variation in the time between ICH and GBS onset. The most common manifestation of GBS associated with ICH was paresis (23/23), consistently accompanied by hyporeflexia or areflexia (23/23), sensory loss or paresthesia (9/23), peripheral facial nerve palsy (7/23), bulbar palsy (4/23-dysphagia &dysarthria, 2/23-not specified), autonomic dysfunction (6/23), and extraocular involvement including ptosis, diplopia, or ophthalmoplegia (9/23). The median time to nadir from GBS onset was 2.5 days (range 0.5–12 days). Clinical severity was assessed using the Hughes Functional Grading Scale with an average score of 4.57 ± 0.66 (range: 3–6) (19, 20).

### Investigations

Electrophysiological examinations were performed in 21 patients, with detailed reports available for eight (Table 2). Of the 23 cases, 26.1% (6/23) were consistent with acute inflammatory demyelinating polyneuropathy (AIDP), while axonal variants accounted for 52.2% (12/23), including five cases of acute motor and sensory axonal neuropathy (AMSAN), five cases of acute motor axonal neuropathy (AMAN), and two unspecified axonal subtypes. In the remaining five cases, the electrophysiological subtype was not reported.

Cerebrospinal fluid (CSF) analysis was conducted in 21 of the 23 cases. Excluding missing data, the average CSF protein level was 222.0 ± 147.7 mg/dL (range: 65–560 mg/dL). Albuminocytologic dissociation (cell count ≤ 5/μL with protein > 45 mg/dL) was reported in 14 of the 22 cases. Mild pleocytosis (cell count > 5/μL) was observed in 7/21 (33.3%) cases, with a maximum cell count of 25/μL. Xanthochromia was reported in one case.

Most cases did not have a documented infection based on the initial workup. Among the five cases tested for specific antibodies, none demonstrated evidence of *C. jejuni* or viral serology. In other cases, an infection was ruled out based on the absence of relevant history, clinical symptoms (fever, diarrhea, respiratory issues) and normal lab results.

Out of the fifteen patients who underwent antiganglioside antibody testing, five tested positive including anti-GM1 (3 cases), GD1a (3 cases), and GD1b (4 cases). Additionally, MRI findings suggestive of GBS such as diffuse contrast enhancement, hyperintense signal changes, and patchy enhancement of the spinal roots were observed in three patients.

### Treatment and prognosis of GBS

The clinical course was severe, with 18 of 23 patients (78.3%) requiring mechanical ventilation for respiratory failure (Tables 3, 4). A total of 21 patients received immunomodulatory treatment: 15(71.4%) were treated with intravenous immunoglobulin (IVIG) alone, 3(14.3%) were treated with plasmapheresis alone, 2(9.5%) with a combination of IVIG and plasmapheresis, and 1(4.8%) with IVIG and methylprednisolone. Two patients did not receive any form of immunomodulatory therapy, and both died. Follow-up durations ranged from 5 weeks to 1 year. At final follow-up, three patients (13%) had fully recovered, thirteen (56.5%) had partial recovery, three (13.0%) had poor recovery, and four (17.4%) had died. Reported causes of death included severe dysautonomia, cardiorespiratory arrest, and infectious complications. Three of the four patients who died had documented sepsis and one met SIRS criteria without a confirmed pathogen, whereas none of the three patients with poor recovery had documented sepsis or SIRS.

**Table 4:** Treatment and prognosis of GBS.

Immunomodulatory treatment	Complete recovery (*n*)	Partial recovery (*n*)	Poor recovery (*n*)	Death (*n*)
IVIG (*n* = 15)	1	11	1	2
PP (*n* = 3)	1	2	0	0
IVIG + PP (*n* = 2)	1	0	1	0
IVIG + methylprednisolone (*n* = 1)	0	0	1	0
No Immunomodulatory treatment (*n* = 2)	0	0	0	2

GBS – Guillain-Barré Syndrome; IVIG – Intravenous Immunoglobulin; PP – Plasmapheresis. Definitions: 1. Complete recovery: The patient has returned to normal function and has no residual deficits. 2. Partial recovery: The patient has recovered some function but still has some residual deficits. 3. Poor recovery: The patient has not recovered much function and has significant residual deficits. This can include being bedridden, requiring assistance with activities of daily living, or being unable to return to work. No recovery: The patient has not recovered any function and remains completely dependent on others for care.

Ganglioside antibody–positive patients (*n* = 5) had markedly worse outcomes than antibody-negative patients (*n* = 10). None of the antibody-positive patients achieved full recovery (2 had poor recovery, 2 partial recovery, and 1 had no recovery and died), whereas 80% of the antibody-negative group showed favorable outcomes, with 1 achieving full recovery and 7 achieving partial recovery (2 died).

A subgroup analysis based on electrophysiological variant (Supplementary Table 1) showed that axonal GBS (AMAN/AMSAN/Axonal NOS, *n* = 12) was associated with a higher need for mechanical ventilation (10/12) compared to demyelinating GBS (*n* = 6; 4/6). Outcomes were less favorable in the axonal group, with only one complete recovery, seven partial recoveries, one poor recoveries, and two deaths. In contrast, demyelinating GBS demonstrated relatively better outcomes, with three complete recoveries, two partial recoveries, one poor recovery, and one death. Within axonal subtypes, AMSAN (*n* = 5) had the poorest prognosis, with no complete recoveries and three cases of poor recovery, whereas AMAN (*n* = 5) showed more favorable outcomes, with one complete recovery and the remainder achieving partial recovery without deaths.

### Quality assessment

To evaluate the methodological quality of studies reporting GBS following ICH, we conducted a JBI quality assessment of case reports (Table 5). Of the 23 case reports identified, 21 were determined to be of high-quality reporting, scoring 7 or 8. However, two studies scored 5, indicating minor issues with reporting. Overall, the assessment findings suggest that the case reports included in our systematic review are of high methodological quality, which enhances the credibility and dependability of our results.

**Table 5:** Quality assessment of included case reports using Joanna Briggs Institute (JBI) critical appraisal checklist for case reports.

S. no	Reference (author, year)	A1	A2	A3	A4	A5	A6	A7	A8	Total
1	Liu et al. (2022) (40)	Yes	Yes	Yes	Yes	Yes	Yes	NA	Yes	7
2	Abdulmana et al. (2021) (14)	Yes	Yes	Yes	Yes	Yes	Yes	NA	Yes	7
3	Abdulmana et al. (2021) (14)	Yes	Yes	Yes	Yes	Yes	Yes	NA	Yes	7
4	Yonekura et al. (2018) (18)	Yes	Yes	Yes	Yes	Yes	Yes	NA	Yes	7
5	Duncan et al. (1987) (41)	Yes	Yes	Yes	No	No	Yes	NA	Yes	5
6	Yilmaz et al. (2020) (42)	Yes	Yes	Yes	Yes	Yes	Yes	NA	Yes	7
7	Connor et al. (2021) (27)	Yes	Yes	Yes	Yes	Yes	Yes	Yes	Yes	8
8	Joshi et al. (2019) (43)	Yes	Yes	No	Yes	Yes	No	NA	Yes	5
9	Wu et al. (2016) (44)	Yes	Yes	Yes	Yes	Yes	Yes	NA	Yes	7
10	Wu et al. (2016) (44)	Yes	Yes	Yes	Yes	Yes	Yes	NA	Yes	7
11	Carr et al. (2015) (15)	Yes	Yes	Yes	Yes	Yes	Yes	NA	Yes	7
12	Mantero et al. (2013) (16)	Yes	Yes	Yes	Yes	Yes	Yes	NA	Yes	7
13	Riancho et al. (2013) (45)	Yes	Yes	Yes	Yes	Yes	Yes	NA	Yes	7
14	Battaglia et al. (2013) (46)	Yes	Yes	Yes	Yes	Yes	Yes	NA	Yes	7
15	Yardimci et al. (2009) (47)	Yes	Yes	Yes	Yes	Yes	Yes	NA	Yes	7
16	Tan et al. (2010) (48)	Yes	Yes	Yes	Yes	Yes	Yes	NA	Yes	7
17	Rivas et al. (2008) (17)	Yes	Yes	Yes	Yes	Yes	Yes	NA	Yes	7
18	Freitas et al. (1997) (49)	Yes	Yes	Yes	Yes	Yes	Yes	NA	Yes	7
19	Hu et al. (2020) (50)	Yes	Yes	Yes	Yes	Yes	Yes	NA	Yes	7
20	Song et al. (2012) (51)	Yes	Yes	Yes	Yes	Yes	Yes	NA	Yes	7
21	Hantson et al. (2013) (13)	Yes	Yes	Yes	Yes	Yes	Yes	NA	Yes	7
22	Jia et al. (2017) (52)	Yes	Yes	Yes	Yes	Yes	Yes	NA	Yes	7
23	Jia et al. (2017) (52)	Yes	Yes	Yes	Yes	Yes	Yes	NA	Yes	7

Answer Options include—Yes, No, Unclear, Not Applicable (NA). A1—Were patient’s demographics characteristics clearly described? A2—Was the patient’s history clearly described and presented as a timeline? A3—Was the current clinical condition of the patient on presentation clearly described? A4—Were diagnostic tests or assessment methods and the results clearly described? A5—Was the intervention(s) or treatment procedure(s) clearly described? A6—Was the post-intervention clinical condition clearly described? A7—Were adverse events (harms) or unanticipated events identified and described? A8—Does the case report provide takeaway lessons?

## Discussion

This systematic review of 23 case reports describes a temporal relationship between antecedent ICH and the subsequent onset of GBS. While the evidence is limited to case-level data and is insufficient to prove causation, this association has been documented following all major types of ICH. The clinical course described in these reports was typically severe, characterized by the rapid onset of symmetrical weakness and a swift progression to maximum deficit. Symptom onset varied from 2 to 24 days post-ICH, a timeframe consistent with the typical presentation of GBS (21). In all cases, the diagnosis was subsequently confirmed through a combination of clinical, cerebrospinal fluid, and electrophysiological findings. Although based on a limited number of cases, these observations underscore the importance of considering GBS in the differential diagnosis when patients with ICH exhibit unexplained, progressive neurological decline—particularly in the absence of new radiographic findings.

A striking finding of our review is the marked severity of GBS in the context of ICH. Approximately three-quarters of patients required mechanical ventilation, compared to the 25–35% typically reported in general GBS populations (22). This severity was further underscored by a rapid progression to nadir and a predominance of axonal GBS subtypes, which are known to be associated with more severe disease and poorer outcomes (23, 24). Our subgroup analysis further supports this, demonstrating that axonal variants were associated with higher rates of mechanical ventilation and worse functional outcomes compared to demyelinating forms, with AMSAN showing the poorest prognosis. This pattern is consistent with prior literature showing that axonal variants, particularly AMSAN, are characterized by rapid progression, increased risk of respiratory failure, and prolonged recovery with greater residual disability (23, 25, 26). The combination of this electrophysiological pattern and the elevated mortality rate, suggests that GBS following ICH may represent a particularly aggressive form of the disease. This likely reflects a multifactorial process involving the severity of the initial brain injury, the superimposed burden of GBS, and the challenges of managing two concurrent, life-threatening neurological conditions.

Most patients received standard immunomodulatory therapy, predominantly intravenous immunoglobulin (IVIG). However, prognosis remained highly variable: around one-fifth of patients died and many survivors experiencing only partial recovery. Interpreting disease severity and treatment response in this population is challenging due to the confounding impact of the initial hemorrhage. Nearly half of the patients had substantial pre-existing neurological deficits from the hemorrhage, and over a quarter required surgical intervention. These factors likely contributed significantly to poor outcomes, independent of the GBS or its treatment. Furthermore, systemic infection was a uniform finding in all fatal cases; three of the four deaths had documented sepsis, while the fourth met SIRS criteria. In contrast, none of the three patients with poor recovery had documented sepsis or SIRS, implying that outcomes in this subgroup were more likely driven by the severity of the initial hemorrhage, the GBS disease burden itself, or both.

While our review describes temporal association, the biological mechanism connecting ICH to GBS remains a subject of speculation. The prevailing theory, analogous to post-infectious GBS, involves molecular mimicry initiated by a breach in the blood–brain barrier (BBB) (24). Following an ICH, the physical disruption of the BBB and the direct cellular damage caused by iron-rich heme released from the lysing hematoma may allow previously sequestered neuronal antigens such as gangliosides and myelin basic protein to enter the systemic circulation (27). This exposure could trigger an autoimmune response that cross-reacts with structurally similar components of peripheral nerves. The presence of anti-ganglioside antibodies (anti-GM1, GD1a, and GD1b) in some patients supports this hypothesis. However, this explanation is not entirely sufficient, as many patients in the reviewed cases tested negative for these specific antibodies. While this does not disprove the molecular mimicry theory, it suggests that alternative or complementary mechanisms may be at play.

An alternative or complementary hypothesis centers on the profound systemic inflammatory response triggered by the initial ICH. A severe brain hemorrhage is a potent inflammatory event, known to induce a “cytokine storm” that releases pro-inflammatory mediators like TNF-*α* and various interleukins into the circulation (28). This inflammatory cascade is likely initiated at the site of the bleed, where the breakdown of hemoglobin releases cytotoxic heme, a powerful pro-oxidant and pro-inflammatory molecule. This systemic inflammation preferentially targets the peripheral nervous system, as its blood-nerve barrier (BNB) is more susceptible to inflammatory breakdown than the resilient blood–brain barrier (BBB) (29). A compromised BNB allows for a direct T-cell mediated attack or inflammatory damage to the peripheral nerves (29), providing a plausible mechanism for GBS development, particularly in the antibody-negative cases observed in our review. It is therefore conceivable that these mechanisms are not mutually exclusive; the ICH could provide the antigenic trigger for molecular mimicry, while the resulting systemic inflammation breaks down the protective BNB, creating a “perfect storm” for autoimmune peripheral nerve injury. It is crucial to note that in most cases, a preceding infection was not identified, strengthening the hypothesis that the ICH itself served as the primary immunological trigger.

Critical illness polyneuropathy (CIP) is an important differential to consider in this context (Table 6) (30). Although CIP is classically described as subacute in onset following prolonged ICU admission, early-onset cases have been documented in patients with systemic inflammatory response syndrome or sepsis, sometimes within the first days of critical illness (31, 32), meaning that temporal profile alone cannot reliably exclude CIP in this setting. From an electrodiagnostic standpoint, AIDP can be distinguished from CIP by demyelinating features such as prolonged compound muscle action potential (CMAP) latency and slowed conduction velocity, while AMAN is characterized by pure motor involvement. Differentiating CIP from AMSAN, however, is more challenging, as both conditions demonstrate reduced CMAP and sensory nerve action potential (SNAP) amplitudes. In such cases, clinical features and ancillary testing become essential. AMSAN more frequently involves cranial nerves, particularly the facial nerve, and is often associated with dysautonomia, findings not typical of CIP. It also commonly demonstrates albuminocytologic dissociation in CSF and may show nerve root enhancement on MRI, whereas CIP does not. Intravenous immunoglobulins (IVIG) and plasma exchange can be effective in AMSAN (25, 33), but not in CIP, aside from limited reports in severe COVID-19 (34).

**Table 6:** Differentiation between critical illness polyneuropathy and Guillain–Barré syndrome.

	Critical illness polyneuropathy	Guillain–Barré syndrome	
		AIDP	AMAN and AMSAN
Prodromal conditions	Sepsis and multiple organ failure	Gastrointestinal or respiratory infection	
Clinical presentation	Onset of the disorder usually after intensive care unit admission Often characterized by fairly symmetric limb muscle weakness sparing cranial nerves; sensory deficits less prominent	Onset of the disorder is usually before intensive care unit admission. Infections precede the onset of progressive weakness and sensory disturbances; frequent cranial nerve involvement and dysautonomia. Sensory symptoms are present in AIDP and AMSAN. Axonal variants can have a more rapid progression leading to paralysis and respiratory failure over a few days.	
Cerebrospinal fluid	Usually normal	Albuminocytologic dissociation	
Electrophysiology	Axonal motor & sensory polyneuropathy	Demyelinating polyneuropathy	Axonal motor polyneuropathy in AMAN, axonal motor & sensory polyneuropathy in AMSAN. It can have unresponsive nerves, abundant spontaneous activity.
Magnetic resonance imaging	No significant findings	Occasional enhancement of spinal nerve roots	
Biopsy	Primarily axonal degeneration of distal peripheral nerves without inflammation	Primarily demyelinating process with inflammation	Motor/sensory axonal degeneration, or motor axonal degeneration only.
Treatment	No specific therapy, usually anti-septic treatment	Plasmapheresis, intravenous immune globulin	
Outcome	Recovery may be spontaneous and of variable timing; 50% of patients with full recovery	Usually > 60% complete recovery	AMAN: Some recover rapidly, others slowly depending on axonal damage. AMSAN: prolonged course with greater residual disability.

*AIDP: Acute inflammatory demyelinating polyneuropathy, AMAN: Acute motor axonal neuropathy, AMSAN: Acute motor-sensory axonal neuropathy. Information compiled from Zhou et al. (30).

Taken together, several findings argue against CIP in the case reports of this systematic review. First, the clinical picture was typical of GBS with rapidly progressive symmetric ascending weakness and generalized areflexia, often with cranial or autonomic involvement, which is not characteristic of CIP (35). Second, the diagnostic studies supported GBS. CSF consistently showed albuminocytologic dissociation, and nerve conduction studies predominantly showed patterns consistent with AIDP. Third, the presence of antiganglioside antibodies in a subset of cases supports an immune mediated process instead of the metabolic and microvascular derangements of critical illness (35).

We acknowledge that intracranial hemorrhage particularly subarachnoid and intracerebral subtypes along with neurosurgical procedures and traumatic lumbar punctures can introduce blood into the CSF (36, 37). Such contamination may spuriously raise protein, and cell counts. However, a substantial RBC burden is required to produce a clinically meaningful false elevation (approximately 1 mg/dL of protein and about 1 WBC per 500–1,000 RBCs) (38). Among the included cases, only one case reported xanthochromia, while CSF analysis was precluded in two others due to procedural deferral or grossly bloody fluid. Although the absence of precise RBC counts in multiple cases prevents the definitive exclusion of blood-related distortion, the consistent observation of albuminocytologic dissociation without significant pleocytosis serves as supportive evidence for GBS in this context.

The quality assessment of the reports included revealed that most were of high methodological quality, which strengthens the reliability of our findings. However, our study has certain limitations that must be considered. First, our study only included case reports, which are subject to publication bias, potentially favoring the reporting of more severe or unusual cases, while milder or self-limited cases may remain unpublished. This may lead to an overestimation of true disease severity and mortality. Second, the lack of a control group precludes the establishment of causality or the calculation of true incidence, limiting our findings to a temporal association. Third, the largest proportion of cases in our review originated from China, where axonal GBS variants are more frequently reported than in many western populations (39). This geographic clustering may have contributed to the high proportion of axonal phenotypes observed and limits the generalizability of our electrophysiological findings to regions in which AIDP predominates. Fourth, the retrospective nature of the data resulted in heterogeneous and inconsistent reporting of key clinical and radiographic details, including the specifics of electrophysiological studies, the use of standardized electrodiagnostic criteria (e.g., EAN/PNS criteria), the inconsistent performance of antiganglioside antibodies testing, and the thoroughness of the workup to exclude antecedent infections. Furthermore, variations in the reporting of hemorrhage severity, anatomical characteristics, standardized grading scales, and radiographic features such as mass effect or midline shift may have independently influenced neurological recovery and mortality, confounding the interpretation of GBS related outcomes.

Despite these limitations, our study offers several valuable insights. From a clinical standpoint, this review highlights the significant diagnostic challenge of “diagnostic overshadowing.” In a patient recovering from a severe brain injury, new-onset weakness can easily be misattributed to the primary injury, sedation, or critical illness myopathy/neuropathy. Recognizing the symmetrical, ascending pattern of weakness, associated hyporeflexia or areflexia, and confirming the diagnosis through CSF analysis (albuminocytologic dissociation) is therefore critical. Although both patients who did not receive immunotherapy died, this cannot be attributed to treatment alone given the concurrent confounders of hemorrhage severity, location, and systemic complications such as sepsis. Nonetheless, prompt initiation of IVIG or plasmapheresis remains reasonable once GBS is suspected, as these represent standard of care and diagnostic delay carries independent risk.

## Conclusion

GBS is a rare but clinically significant complication that can occur following an ICH. Our analysis suggests that post-ICH GBS represents a particularly aggressive clinical phenotype, characterized by rapid progression to nadir and a high rate of respiratory failure. These findings underscore the critical need for clinicians to maintain a high index of suspicion for GBS in patients with ICH who develop progressive, symmetrical weakness unexplained by the initial brain injury. While prompt diagnosis and initiation of standard immunotherapy is appropriate once GBS is suspected, outcomes in this population are likely multifactorial, and the independent contribution of treatment cannot be established from case-level data alone. Future research should focus on prospective, multi-center registry-based studies to determine the true incidence of this association and to conduct more systematic immunological analyses to elucidate the precise pathophysiological link between CNS hemorrhage and peripheral nervous system autoimmunity.

## Data Availability

The original contributions presented in the study are included in the article/supplementary material, further inquiries can be directed to the corresponding author.
