# Supplementary material for: Guillain-Barré syndrome following intracranial hemorrhage: a systematic review of case reports
Source: Front Neurol. 2026 Jun 17;17:1789597. doi: 10.3389/fneur.2026.1789597 (PMC13321458; doi:10.3389/fneur.2026.1789597)
Supplement: Supplementary file 1 [file Table_1.docx]

**Supplementary table 1:** Electrophysiological variant–based subgroup analysis of treatment outcomes and prognosis in GBS.

| **Variant** | **n** | **Mechanical ventilation** | **Immunomodulatory Treatment (IVIG/PP)** | **Complete recovery (n)** | **Partial recovery (n)** | **Poor recovery (n)** | **Death (n)** |
| --- | --- | --- | --- | --- | --- | --- | --- |
| Axonal GBS (AMAN/AMSAN/Axonal NOS) | 11 | 7 | 10 | 1 | 6 | 3 | 2 |
| Demyelinating GBS (AIDP/demyelinating NOS) | 7 | 1 | 6 | 3 | 2 | 1 | 1 |
| AMSAN only | 5 | 3 | 5 | 0 | 2 | 3 | 0 |
| AMAN only | 5 | 3 | 5 | 1 | 4 | 0 | 0 |

**Abbreviations:** GBS, Guillain-Barré syndrome; AIDP, acute inflammatory demyelinating polyneuropathy; AMAN, acute motor axonal neuropathy; AMSAN, acute motor and sensory axonal neuropathy; IVIG, intravenous immunoglobulin; PP, plasmapheresis; NOS, not otherwise specified.
